# Supplementary material for: Histone variant H2A.J accumulates in senescent cells and promotes inflammatory gene expression
Source: Nat Commun. 2017 May 10;8:14995. doi: 10.1038/ncomms14995 (PMC5436145; doi:10.1038/ncomms14995)
Supplement: Supplementary Information — Supplementary Figures and Supplementary Tables [file ncomms14995-s1.pdf]

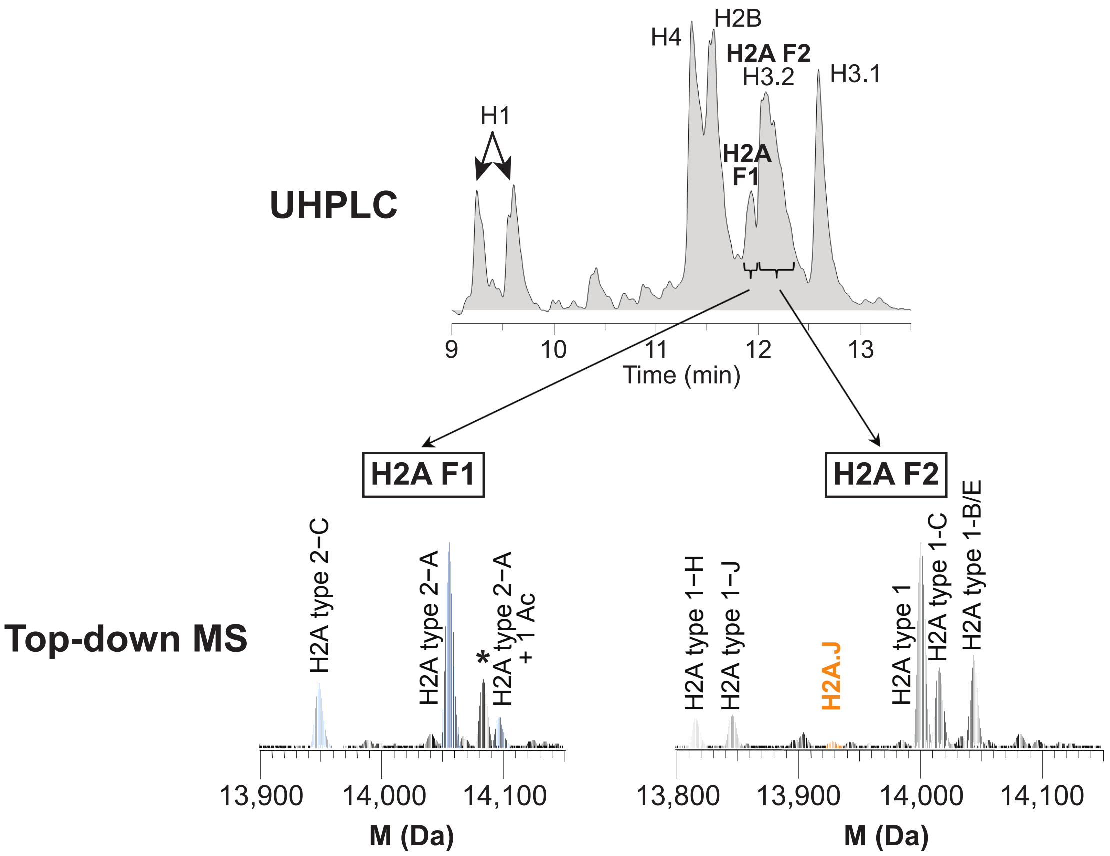

Supplementary Figure 1. Ultra-High Performance Liquid Chromatography-Mass Spectrometry of histones of the two H2A fractions. H2A species with Met52 (H2A types 2-A and 2-C) elute in Fraction 1 (H2AF1) and are indicated as H2A1 in Fig. 1d. H2A species with Leu52 (H2A type 1-H, H2A type 1-J, H2A.J, H2A type 1, H2A type 1-C and H2A type 1-B/E) elute in Fraction 2 (H2AF2) and are indicated as H2A2 in Fig. 1d. See Supplementary Figs. 2,3 for a sequence alignment and phylogeny of human H2A proteins. The top-down mass spectra shown correspond to histones extracted from proliferating WI-38 human fibroblasts. Masses and accuracies of the main human H2A species observed by top-down MS are shown in Supplementary Table 1. * = unidentified protein.

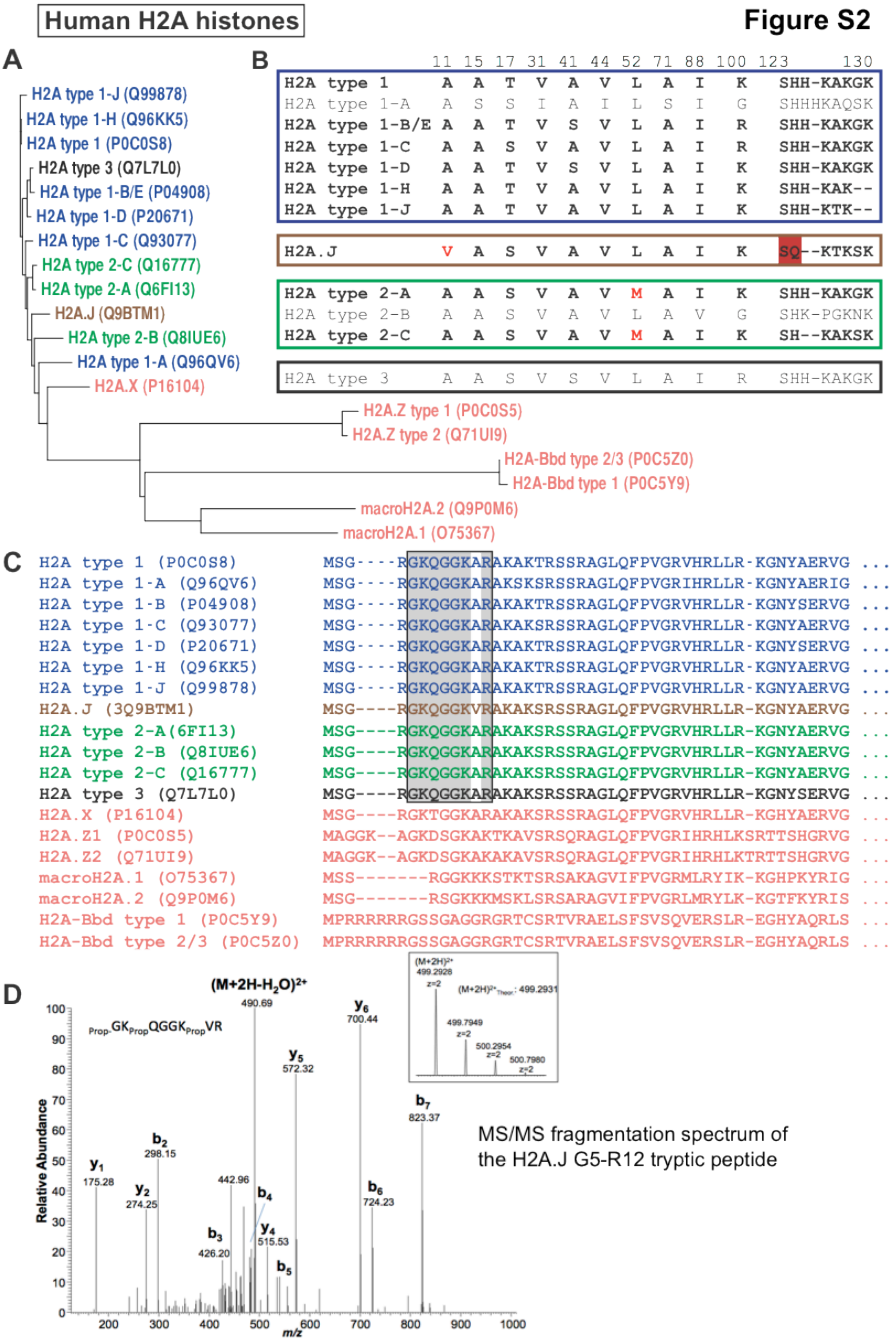

Supplementary Figure 2. **Human H2A histones**. (**A**) Phylogenetic tree of human H2A histones with their accession number (H2A1 in green, H2A2 in blue and H2A.J in brown). (**B**) Sequence alignment showing variation between H2A.J and the canonical H2As. H2A histones that were identified at the protein level by UHPLC-MS (see Supplementary Fig. 1) are indicated in bold. (**C**) H2A amino-terminal sequences are shown with the peptide GKQGGKVR that is unique to H2A.J whereas GKQGGKAR is found in all canonical H2As. These two peptides were used to quantify H2A.J levels relative to all canonical H2As in Fig. 1c. (**D**) MS/MS fragmentation spectrum of the G5-R12 tryptic peptide found in d-SenRep histones that was used to identify and quantify H2A.J. Histones were propionylated before and after trypsin digestion as indicated in the Methods.

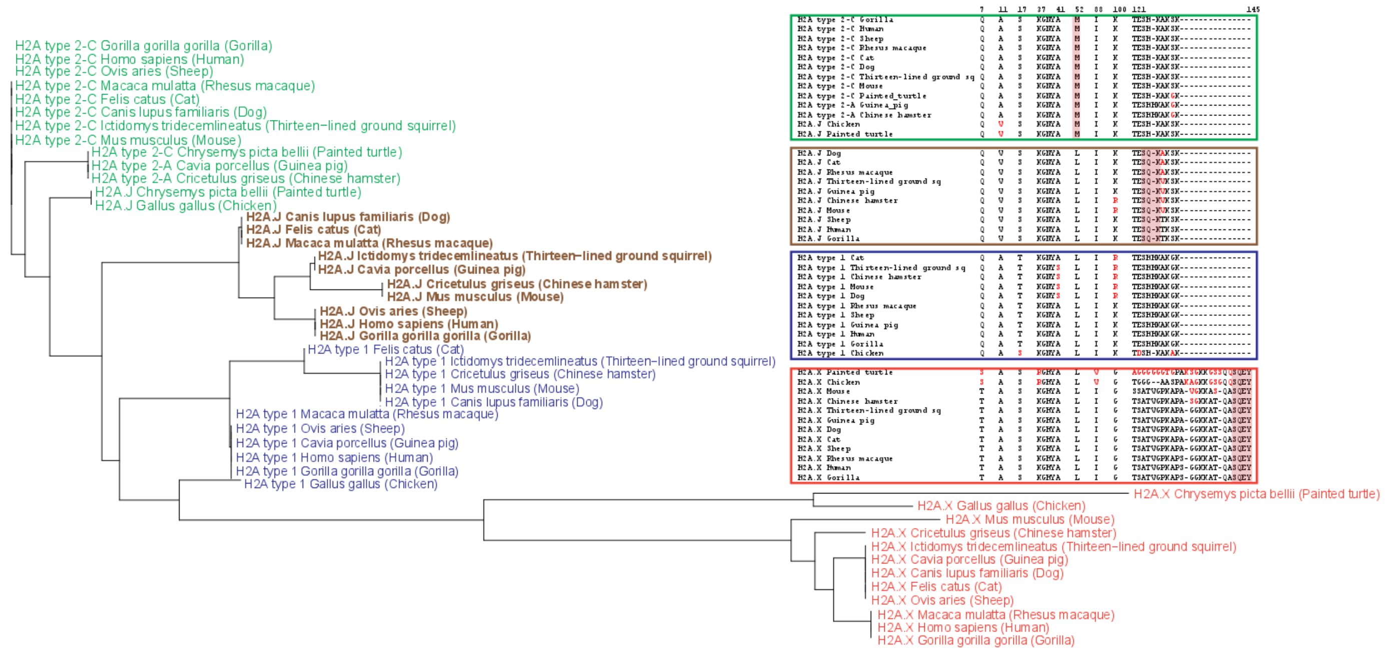

Supplementary Figure 3. Phylogenetic tree showing restriction of the H2A.J histone variant to the mammalian lineage, and sequence alignments of the H2A.J (brown), H2A1 (blue), H2A2 (green), and H2A.X (red) families. The mammalian H2A.J variant is characterized by Val11 and an SQK motif 5 residues from the C-terminus of the protein. The residue at -4 from the C-terminus is hyper-variable in the H2A.J variants: it is Val in the rodent family, Thr in humans, gorilla, sheep, and Ala in most other Mammalia. The canonical H2As (H2A1 and H2A2 families) all contain Ala11 without SQ. The H2A.X variant contains Ala11 and an SQEY motif at the C-terminus of the protein that is 12-15 residues longer than H2A.J and the canonical H2As. Note that a sequence encoded in many bird and reptilian genomes is annotated as H2A.J apparently because of a Val at position 11, but these sequences do not contain a C-terminal SQ motif (see chicken and painted turtle). These sequences also contain Met52 whereas the mammalian H2A.J species all contain Leu52. The mammalian H2A.J family may thus have evolved from an H2A sequence that acquired an A11V mutation during the evolution of the chordates followed by M52L and H124Q mutations during the evolution of the Mammalia.

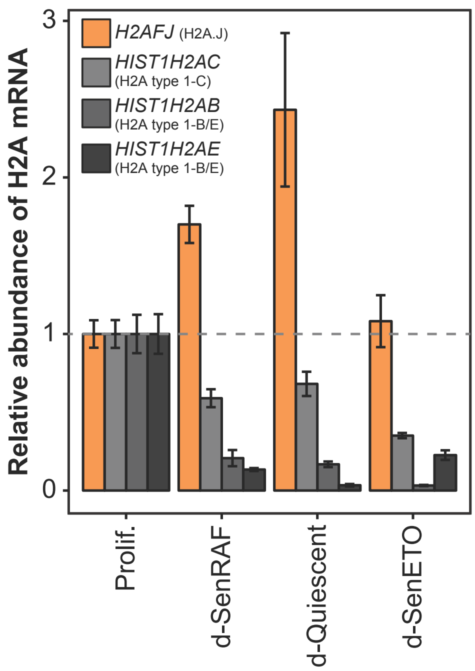

Supplementary Figure 4. **RT-qPCR analysis of RNA levels of *H2AFJ* and selected canonical H2A species in senescent or quiescent cells relative to proliferating cells**. Random primers were used to prepare cDNA to quantify total RNA. This experiment is an independent biological replicate of Fig. 3a. RNA levels were normalized to *GAPDH* mRNA and to the levels of *H2AFJ* mRNA in proliferating fibroblasts. RNAs were extracted from proliferating WI-38hTERT/GFP-RAF-ER fibroblasts (Prolif.), the same cells induced into quiescence by serum starvation for 20 days (d-Quiescent), induced into senescence by the expression of a hyperactive RAF kinase for 20 days (d-SenRAF), or induced into senescence by treatment with etoposide for 20 days (d-SenETO).

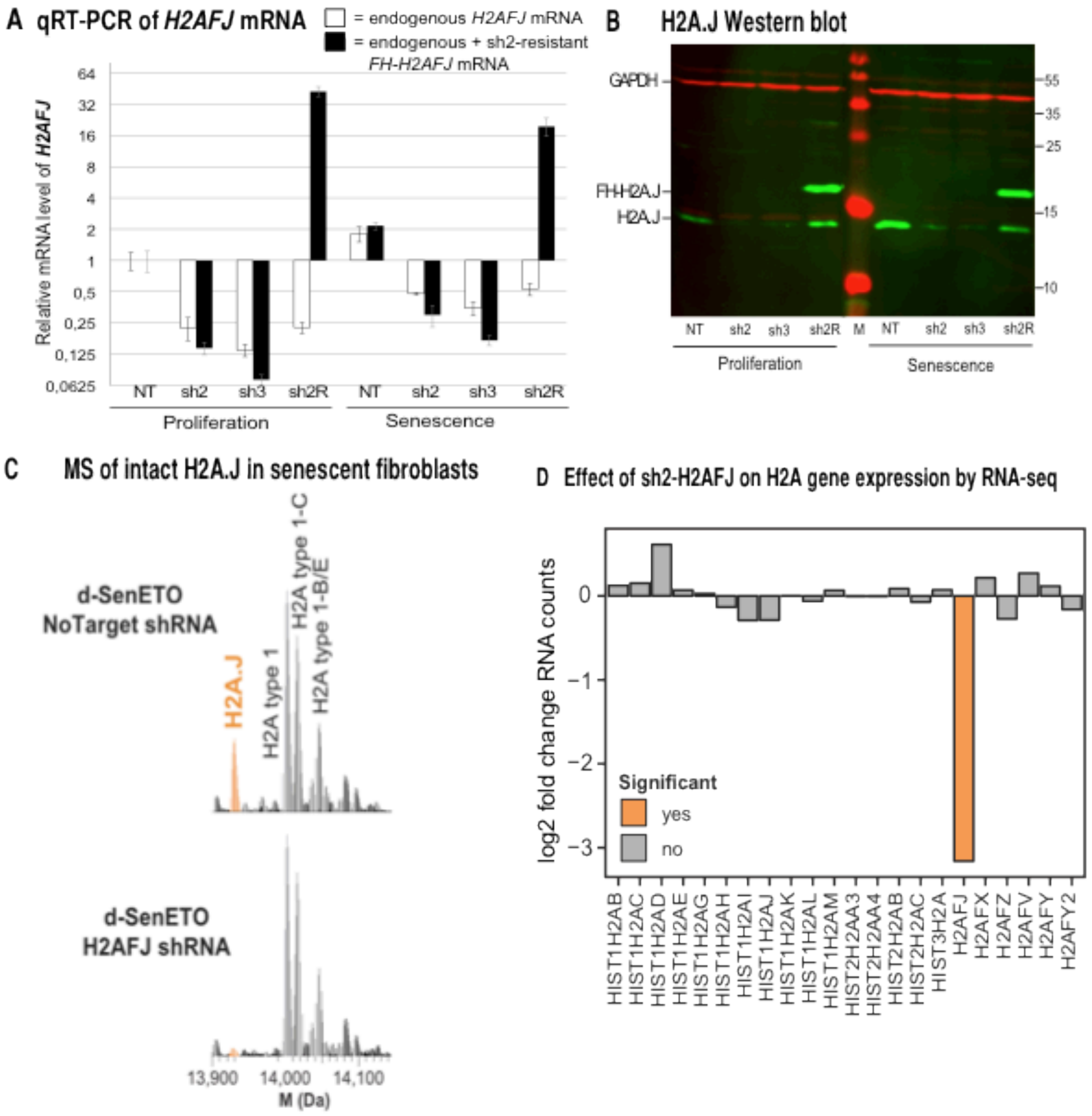

Supplementary Figure 5. Two distinct lentiviral shRNA sequences effectively knock-down *H2AFJ* mRNA and prevent accumulation of H2A.J in senescent human fibroblasts, and expression of an sh2-resistant *H2AFJ* cDNA restores *H2AFJ* expression. (**A**) RT-qPCR analysis of *H2AFJ* mRNA in proliferating and etoposide-senescent WI38hTERT fibroblasts expressing an sh-NoTarget RNA (NT), two different sh-*H2AFJ* RNAs (sh2 and sh3), or the sh2 RNA as well as an sh2-resistant *H2AFJ* mRNA (sh2R). All shRNAs were expressed from pTRIPz. The black boxes show the quantity of total *H2AFJ* mRNA (endogenous + sh2 resistant-Flag-HA-*H2AFJ*) using primers within the *H2AFJ* coding sequence. The white boxes show the quantity of endogenous *H2AFJ* mRNA using a primer that is specific to the 5'-untranslated region of the endogenous *H2AFJ* mRNA. Mean and s.d. for n=3. (**B**) anti-H2A.J (green) Western blot of whole cell extracts from the cells described in (**A**). The sh2-resistant form of *H2AFJ* also contains a Flag-HA epitope fused to the N-terminus of H2A.J (FH-H2A.J). GAPDH (red) was used as a loading control. Note that a band migrating at the level of endogenous H2A.J in cells expressing the sh2-resistant

FlagHA-H2A.J cDNA is apparently due to some proteolysis of the N-terminal Flag-HA tag. (**C**) Deconvoluted top-down mass spectra of H2A2 histones from senescent WI38hTERT fibroblasts expressing sh-No Target or sh2-*H2AFJ* RNAs. (**D**) Effect of sh2-*H2AFJ* on the expression of all related H2A gene sequences in the human genome as determined by RNA-seq. RNA-seq data was obtained from a biological triplicate of WI-38hTERT cells expressing either sh2-*H2AFJ* or sh-NoTarget RNAs and induced into senescence by etoposide treatment for 20 days. The graph shows the log2 fold-change of RNAs for the indicated H2A genes for cells expressing sh2-*H2AFJ* versus sh-NT. Only the *H2AFJ* mRNA is significantly decreased by the expression of sh2-*H2AFJ*.

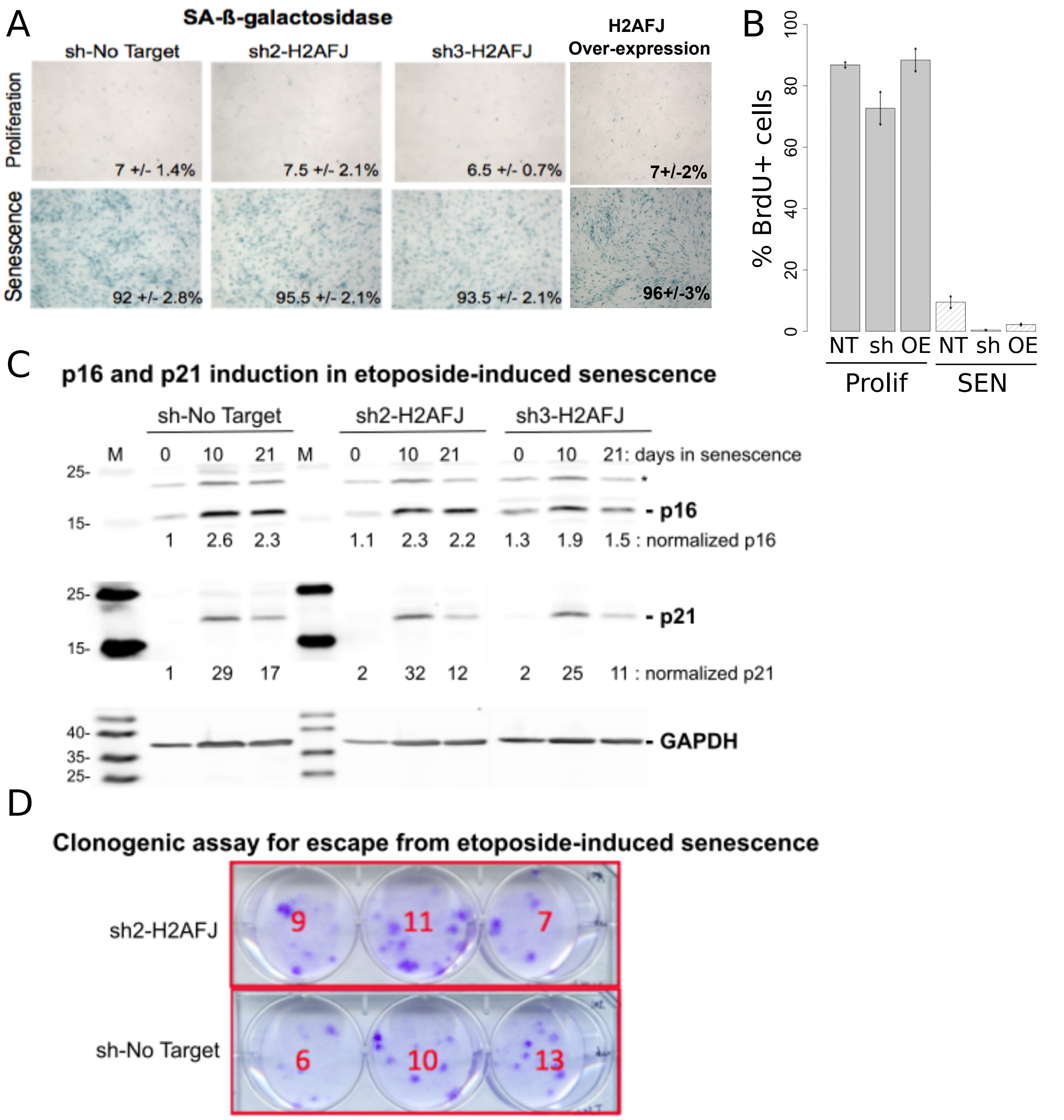

Supplementary Figure 6. H2A.J knock-down or over-expression does not inhibit senescence induction (A,B,C) and H2A.J knock-down does not affect the maintenance of etoposide-induced senescence (D). (**A**) WI-38hTERT fibroblasts expressing sh-No Target, sh2-*H2AFJ*, sh3-*H2AFJ* RNAs, or ectopically over-expressing *H2AFJ*, in proliferation, or after treating with 20 µM etoposide for 1 week, were stained for senescence-associated ß-galactosidase activity. The percentage of positive cells is shown. Mean and sd for 3 fields containing at least 200 cells. (**B**) % cells incorporating BrdU. WI-38hTERT fibroblasts expressing sh-No Target (NT), sh3-*H2AFJ* RNA (sh), or ectopically over-expressing *H2AFJ* (OE), in proliferation (Prolif) or after 6 days of etoposide treatment (SEN) were incubated with BrdU for 24 hours. (**C**) Immunoblot of p16 and p21 expression in the same cells after addition of etoposide for the indicated number of days. *= non-specific band. M=marker. (**D**) WI-38hTERT fibroblasts expressing sh-No Target or sh2-*H2AFJ* RNAs were induced into senescence by treatment with 20 µM etoposide for 2 weeks. Cells were then trypsinized and replated at a density of 10,000 cells per 35 mm dish and incubated for a further 2 weeks without etoposide to allow the proliferation of clones that had escaped senescence. Triplicate replicates of crystal violet stained cells are shown. No significant difference in senescence escape frequency was observed for fibroblasts expressing sh-No Target or sh2-*H2AFJ*.

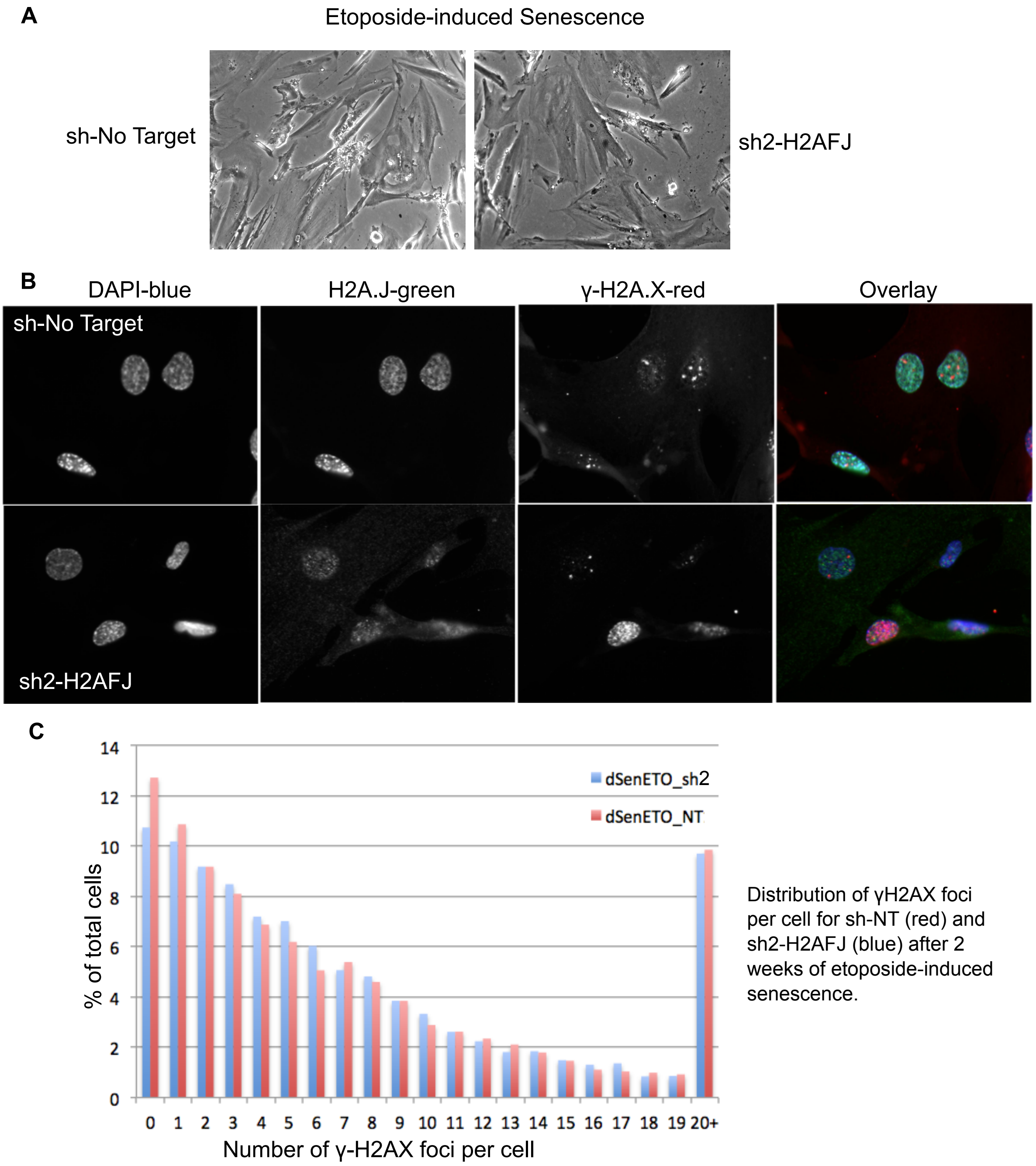

Supplementary Figure 7. **H2A.J depletion does not affect the morphology, DNA compaction, or γH2AX foci distribution of WI-38hTERT fibroblasts in etoposide-induced senescence**. Cells expressing sh-No Target or sh2-*H2AFJ* RNAs were treated with 20 µM etoposide for 2 weeks to induce senescence. (**A**) Phase-contrast imaging of cell morphology. (**B**) Immunofluorescent staining of H2A.J and γH2AX. DNA compaction was visualized with DAPI. (**C**) Quantification of γH2AX foci per cell using an Operetta high-content image analyser.

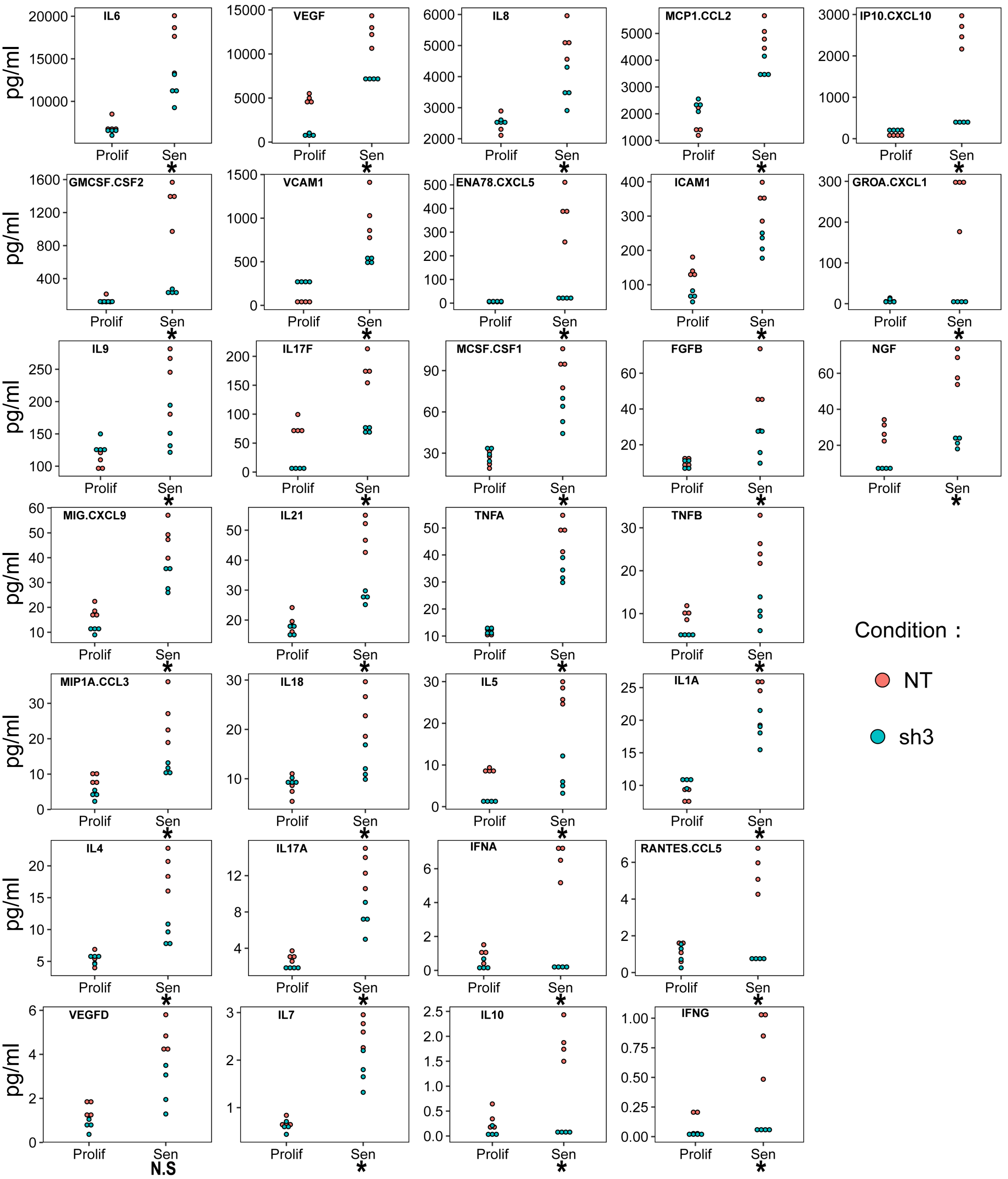

Supplementary Figure 8. **Luminex bead immunoassay of the indicated human cytokines, chemokines, and growth factors in conditioned media**. Dot plots showing the concentration (pg/ml) of the indicated human proteins secreted into the media of WI-38hTERT cells expressing sh-NoTarget RNA (salmon color) or sh3-*H2AFJ* RNA (turquoise color) for cells in proliferation (Prolif) or senescence induced by etoposide treatment (Sen). Each dot represents a biological replicate. An asterisk below the strip-plot indicates a significant difference ($p < 0.05$) between sh-NT and sh3-*H2AFJ* using a t-test with Benjamini & Hochberg correction for multiple tests with the p.adjust function in the R stats package.

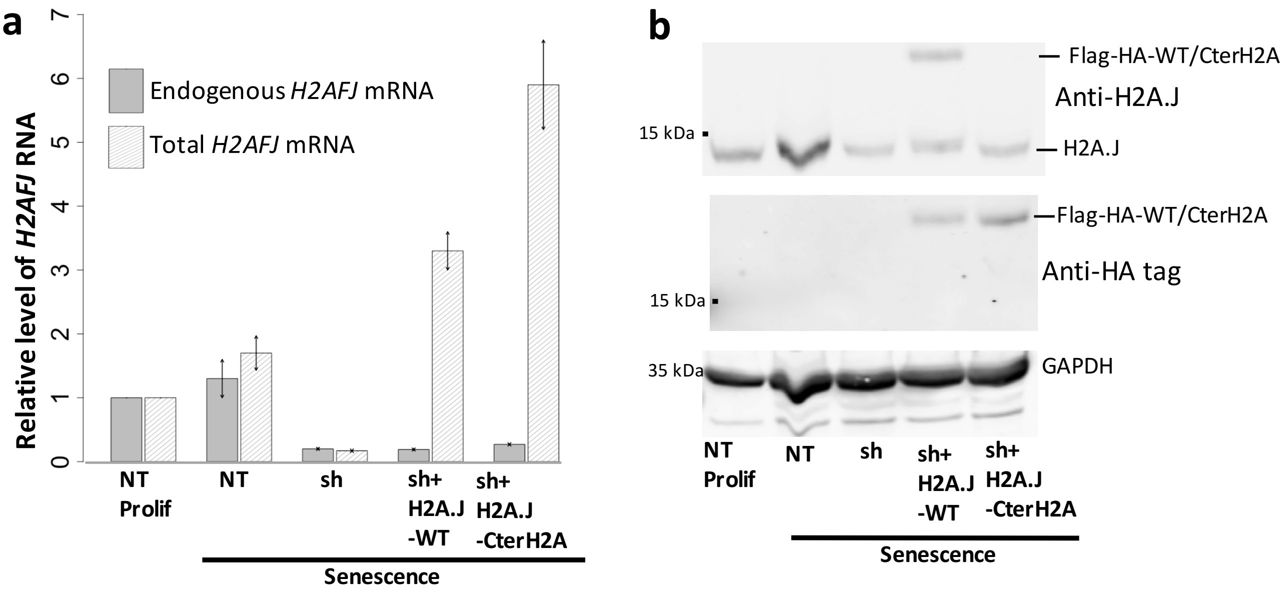

Supplementary Figure 9. Expression levels of endogenous and ectopically expressed *H2AFJ* RNAs and H2A.J proteins in functional complementation experiments.

WI-38/hTERT cells were infected with pGIPz lentiviruses expressing a No Target shRNA (NT) or an sh3-*H2AFJ* RNA (sh). The sh3-*H2AFJ* cell line was super-infected with pTRIPz lentiviruses expressing sh3-resistant *H2AFJ* cDNAs encoding WT-H2A.J or a mutant H2A.J in which the C-terminus of H2A.J was substituted with the C-terminus of canonical H2A type 1 (H2A.J-Cter-H2A). Both the WT and mutant H2A.J contained an N-terminal Flag-HA tag. RNA was isolated from cells induced into senescence by etoposide treatment and compared to levels in proliferating sh-No Target cells. (**a**) RT-qPCR quantification of endogenous *H2AFJ* mRNA (dark grey) and total *H2AFJ* mRNA (endogenous + ectopically-expressed cDNAs encoding N-terminally-tagged Flag-HA-H2A.J WT and H2A.J-Cter-H2A proteins). Results show the mean and s.d. from 3 biological replicates. (**b**) Western blots of total cell extracts from the indicated cells with anti-H2A.J, anti-HA(hemagglutinin) tag, and anti-GAPDH antibodies. Note that the anti-H2A.J Ab directed to the specific C-terminus of H2A.J recognizes the Flag-HA-H2A.J protein but does not recognize the Flag-HA-H2A.J-Cter-H2A protein, whereas both proteins are recognized by the anti-HA tag Ab. Both the RT-qPCR and Western analyses indicate that the Flag-HA-H2A.J-Cter-H2A was mutant was expressed at slightly higher levels than the Flag-HA-H2A.J WT protein.

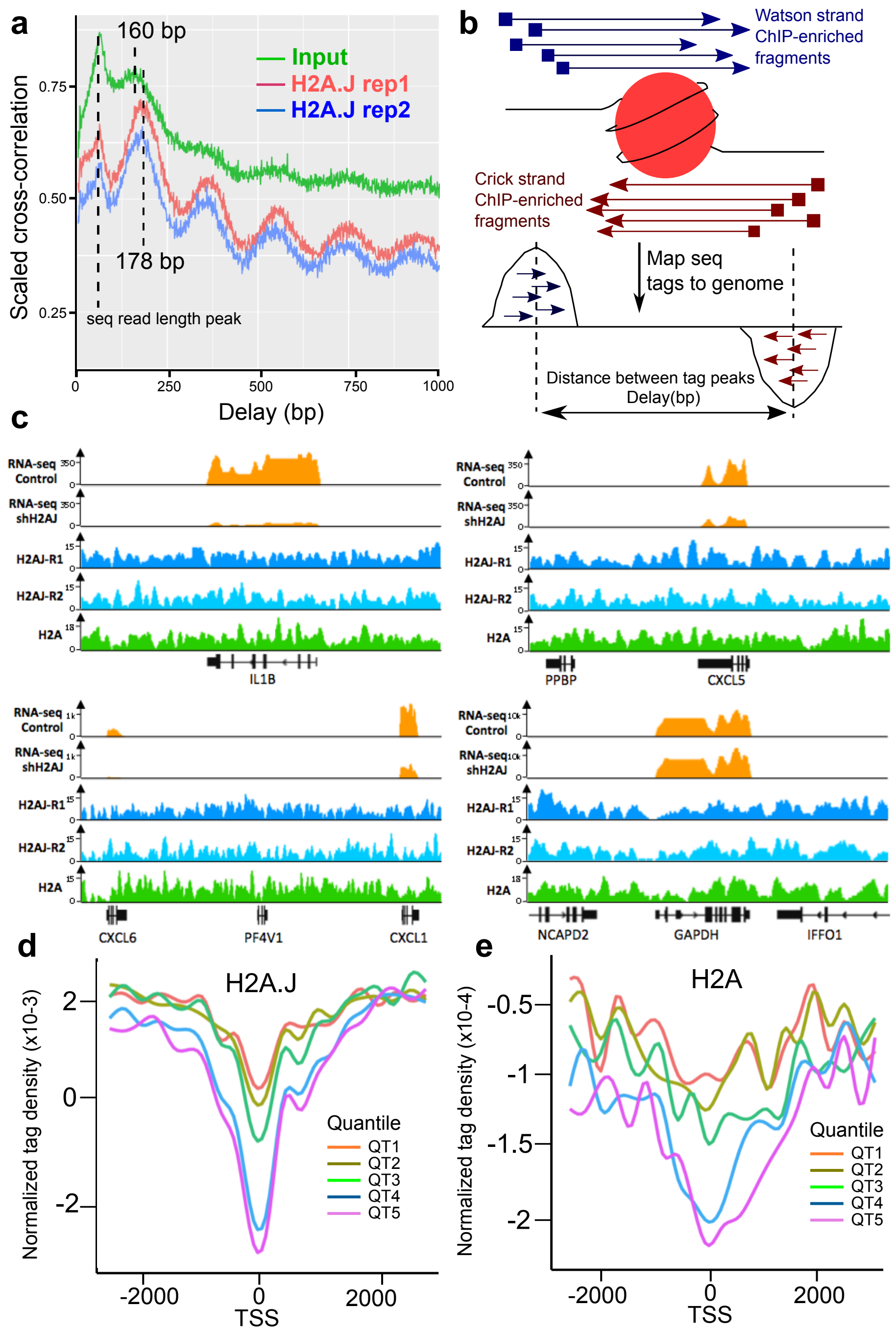

Supplementary Figure 10. ChIP-seq shows that H2A.J is broadly distributed in the genome of senescent cells and depleted at the transcription start sites (TSS) of highly expressed genes. (**a**) Cross-correlation of tag frequency distributions for chromatin immunoprecipitated with anti-H2A.J (red and blue for two replicates) versus Input chromatin (green). Peaks in the tag frequency cross-correlation plot provide information about the characteristic size of the mononucleosome-, di-, tri-nucleosome protected fragments. The smallest peak corresponds to the length of the DNA sequence reads. (**b**) Schema explaining how cross-correlation plots are generated. Reads are shifted in the direction of the strand they map to (Watson in blue, Crick in red) by an increasing number of base pairs and the Pearson correlation between the per-position read count vectors for each strand is calculated. (**c**) Distributions of the sequenced tag density for two replicate ChIP-seq libraries of H2A.J (blue and cyan) and one H2A ChIP-seq library at four genomic loci for cells in etoposide-induced

senescence. The upper tracks (orange) show RNA-seq coverage expressed in RPKM for cells expressing sh-NT (Control) or sh2-*H2AFJ* RNA in etoposide-induced senescence. (**d,e**) Average enrichment profiles around Transcription Start Sites (TSS) calculated for H2A.J and H2A. The genes were placed into five groups of equal sizes according to their expression levels (expression quantiles, with QT1 corresponding to the lowest, QT5 to the highest expression levels) in etoposide-induced senescence and the normalized tag densities were calculated around the TSS.

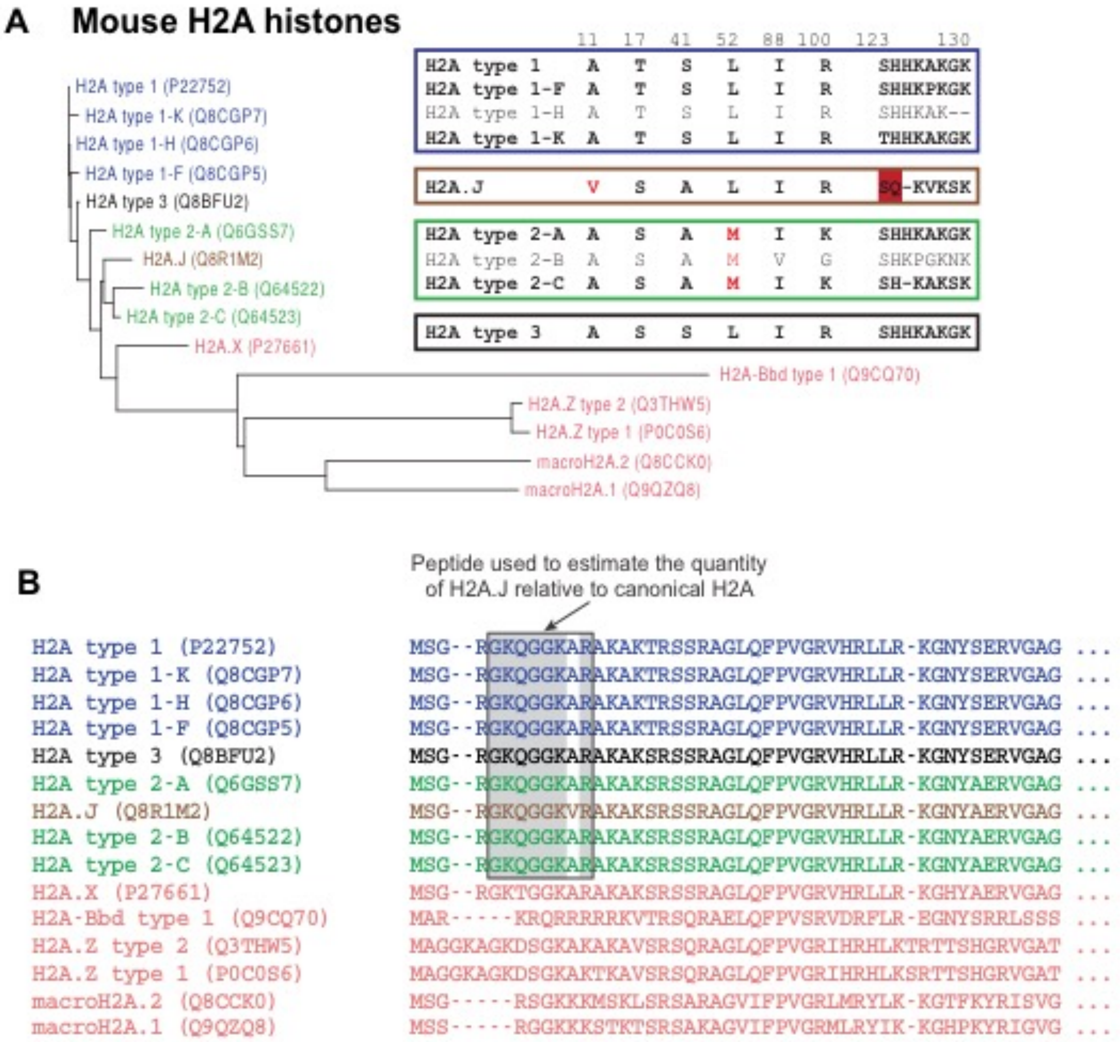

Supplementary Figure 11. **Mouse H2A histones**. (**A**) Phylogenetic tree of all mouse H2A histones with their accession number. Variation in amino acid sequences is shown. H2A histones that were identified at the protein level (Fig. 8) are indicated in bold. See Supplementary Table 2 for a list of masses used to identify these proteins (**B**) H2A amino-terminal sequences are shown with the peptide GKQGGKVR that is unique to H2A.J whereas GKQGGKAR is found in all canonical H2A histones These two peptides were used to quantify H2A.J levels relative to all canonical H2A species in Fig. 8d.

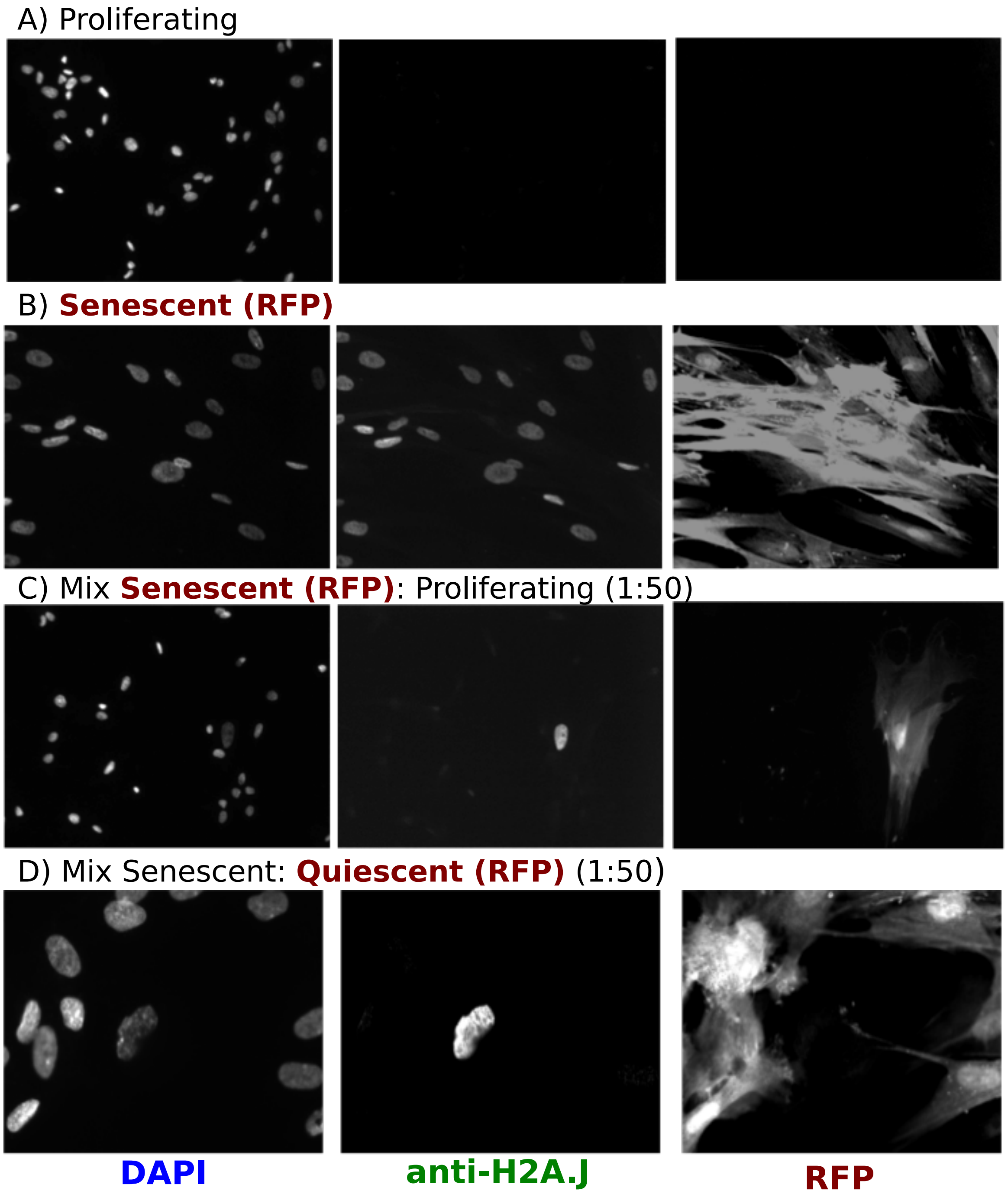

Supplementary Figure 12. **H2A.J immunofluorescence allows detection of senescent human fibroblasts with high sensitivity and specificity in mixing experiments**. WI-38hTERT cells expressing RFP were induced into senescence by treating with 20 µM etoposide for 2 weeks, or incubated with medium containing only 0.1% fetal bovine serum to induce quiescence. Proliferating fibroblasts without RFP, senescent RFP cells and a mix of the two at the indicated ratios were fixed and treated for indirect immunofluorescence with rabbit anti-H2A.J antibodies and anti-rabbit-IgG-Alexa488 (green) secondary antibodies. (**A**) Proliferating cells stain weakly for H2A.J (**B**) Senescent-RFP cells stain strongly for H2A.J (**C**) H2A.J staining identifies the rare senescent cells in a mix (1:50) of senescent:proliferating cells. (**D**) Senescent cells without RFP were mixed with quiescent-RFP cells at a ratio of 1:50. H2A.J staining readily distinguished the rare senescent cells from the quiescent cells. Note that panels A-C show a 200x magnification whereas panel D show a 630x magnification.

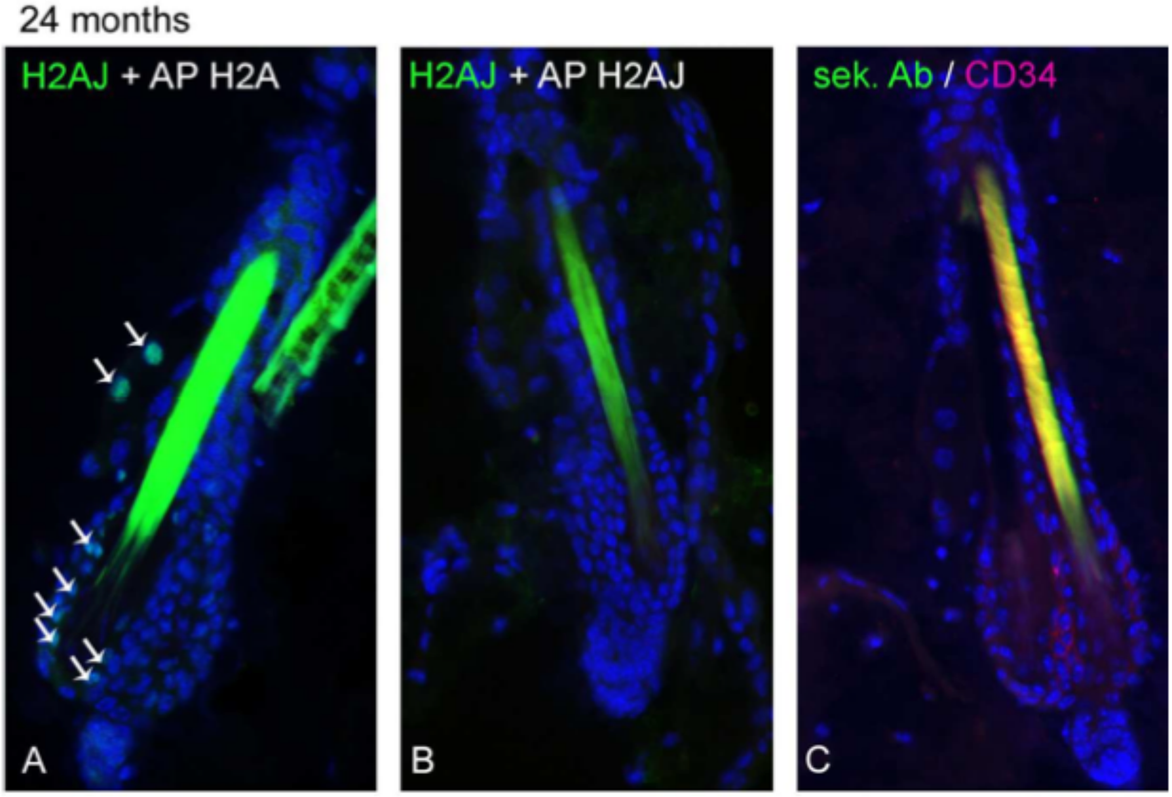

Supplementary Figure 13. **Specificity of the H2A.J immunofluorescent signal demonstrated by competition with antigenic peptides**. Sections of 24-month old mouse skin samples were stained with antibodies to H2A.J in the presence of a 1,000-fold molar excess of the C-terminal peptide of canonical H2A (TESHHKAKGK-panel **A**), or the C-terminal peptide of H2A.J (TESQKTKSK-panel **B**). The H2A.J peptide competed out the signal whereas the canonical H2A peptide had no effect. **C**) The secondary Alexa-488-anti-rabbit Ab (green) alone showed no staining. The CD34 mouse Ab and the Alexa-568-anti-mouse Ab (magenta) shows the specific staining of the CD34+ hair follicle stem cells. The hair in the follicles shows broad auto-fluorescence.

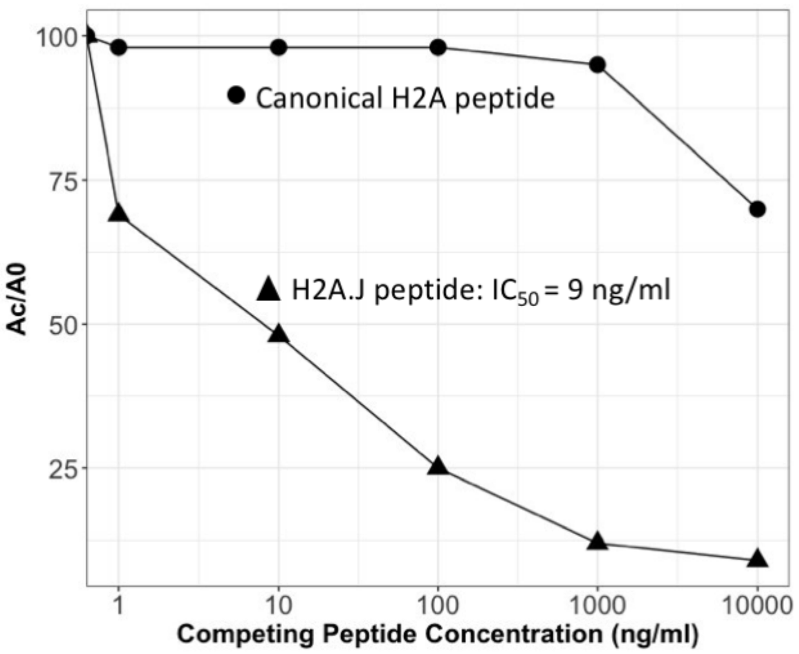

Supplementary Figure 14. Elisa peptide competition assay demonstrating a 1,000-fold higher affinity of the anti-H2A.J antibody for the C-terminal H2A.J peptide compared to a C-terminal canonical H2A peptide. Microplate wells containing the anti-H2A.J Ab were incubated with Acetylcholinesterase (ACE) conjugated to the C-terminal H2A.J peptide in the presence of competing peptides at the indicated concentrations. The binding of ACE-H2A.J to the wells was quantified calorimetrically with Ellman's reagent. Ac/A0 shows the absorbance for binding in presence of the competing peptide at the indicated concentrations divided by the absorbance for binding in the absence of competing peptide. The C-terminal H2A.J peptide (CTESQKTKSK) binds the anti-H2A.J Ab with a 1,000-fold higher affinity than the C-terminal canonical H2A peptide (CTESHHKAKGK). The N-terminal cysteine residues are not present in the histone sequences, but were added to couple the peptides to the ACE enzyme.

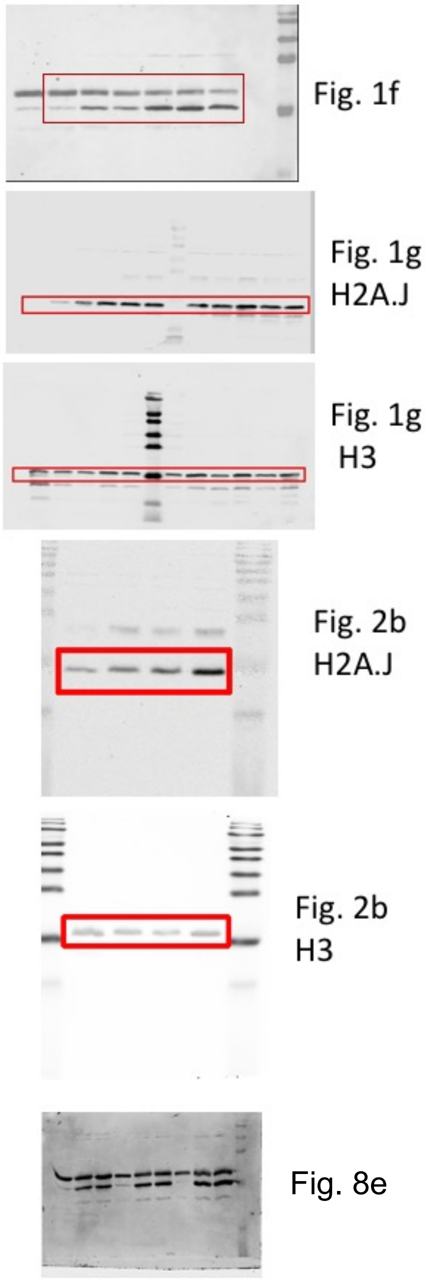

Supplementary Figure 15. Uncropped Western blots.

Supplementary Table 1. Masses and accuracies of the main human H2A species observed by UHPLC-MS and found in a dSenRAF sample as depicted on Fig. 1 and Supplementary Fig. 1. Theoretical and observed masses are monoisotopic masses. Theoretical masses were calculated based on primary sequences and post-translational modifications.

Histones	Accession number	PTMs[a]	Theoretical masses (Da)	Observed masses (Da)	Δm	Error (ppm)
**H2A type 1-H**	Q96KK5	N-terminal acetylation	13,808.80	13,808.68	-0.12	-8.7
**H2A type 1-J**	Q99878	N-terminal acetylation	13,838.81	13,838.88	0.07	5.1
**H2A type 2-C**	Q16777	N-terminal acetylation	13,890.81	13,890.71	-0.10	-7.2
**H2A.J**	Q9BTM1	N-terminal acetylation	13,921.90	13,921.82	-0.08	-5.7
**H2A type 1**	P0C0S8	N-terminal acetylation	13,993.92	13,993.88	-0.04	-2.9
**H2A type 2-A**	Q6FI13	N-terminal acetylation	13,997.86	13,997.87	0.01	0.7
**H2A type 1-C**	Q93077	N-terminal acetylation	14,007.91	14,007.92	0.01	0.7
**H2A type 1-B/E**	P04904	N-terminal acetylation	14,037.92	14,037.86	-0.06	-4.3

[a] Acetylation leads to a mass shift of +42.01 Da.

Supplementary Table 2. Masses and accuracies of the main mouse H2A species observed by UHPLC-MS and found in a kidney extract (3 months) as depicted on Fig. 8. Theoretical and observed masses are monoisotopic masses. Theoretical masses were calculated based on primary sequences and post-translational modifications.

Histones	Accession number	PTMs[a]	Theoretical masses (Da)	Observed masses (Da)	Δm	Error (ppm)
**H2A type 2-C**	Q64523	N-terminal acetylation	13,922.80	13,922.81	0.01	0.7
**H2A.J**	Q8CGP6	N-terminal acetylation	13,947.92	13,947.97	0.05	3.6
**H2A type 3**	Q8BFU2	N-terminal acetylation	14,023.90	14,024.00	0.10	7.1
**H2A type 2-A**	Q6GSS7	N-terminal acetylation	14,029.85	14,029.87	0.02	1.4

**H2A type 1**	P2252	N-terminal acetylation	14,037.92	14,037.95	0.03	2.1
**H2A type1-K**	Q8CGP7	N-terminal acetylation	14,051.94	14,051.95	0.01	0.7
**H2A type 1-F**	Q8CGP5	N-terminal acetylation	14,063.94	14,063.99	0.05	3.6

[a] Acetylation leads to a mass shift of +42.01 D

Supplementary Table 3. Primers used in this study for RT-qPCR experiments:

**Human Primers**		
H2AFJ _EXON1 (1)	for	TCGGGTGCGGTACGTTGCATTC
	rev	GGAGCGGGATTTGGCCTTTGCT
H2AFJ _EXON1 (2)	for	AGCAAAGGCCAAATCCCGCTCC
	rev	CAGCTCCAGGATCTCCGCCGTA
GAPDH	for	ATGGGGAAGGTGAAGGTCG
	rev	GGGGTCATTGATGGCAACAATA
CXCL5	for	AGCTGCGTTGCGTTTGTTTAC
	rev	TGGCGAACACTTGCAGATTAC
CXCL6	for	AGAGCTGCGTTGCACTTGTT
	rev	GCAGTTTACCAATCGTTTTGGGG
IL1A	for	TGGGTTCCCAGTTGGAGTTT
	rev	AGCCTCCTGAATAGCTGGGA
CXCL1	for	AACAGCCACCAGTGAGCTTC
	rev	GAAAGCTTGCCTCAATCCTG
CXCL2	for	GCTTCCTCCTTCCTTCTGGT
	rev	GGGCAGAAAGCTTGTCTCAA
CXCL3	for	ACCCTGCAGGAAGTGTCAAT
	rev	ATCCCCCATGGTTCAGAAA
COL17A1	for	TTACCCGCCATGCGTATGAAG
	rev	CAGTCGAACTCGAATTTCACTCT
CCL2_promoter	for	CCCCTGCTTCCCTTTCCTAC
	rev	TATTGAAAGCGGGCAGAGGG
CCL2_exon	for	CAGCCAGATGCAATCAATGCC
	rev	TGGAATCCTGAACCCACTTCT
IL1B_promoter	for	CGTTGTGCAGTTGATGTCCA
	rev	TGTCTTCCACTTTGTCCCACA
IL1B_exon	for	TTCGACACATGGGATAACGAGG
	rev	TTTTTGCTGTGAGTCCCGGAG
CXCL5_promoter	for	GGAGGAGCGAAGATTGGAG
	rev	CTTTCCACACTGCCCCATGA
CXCL5_exon	for	AGCTGCGTTGCGTTTGTTTAC
	rev	TGGCGAACACTTGCAGATTAC
GAPDH_promoter	for	AAAAGCGGGGAGAAAGTAGG

	rev	AAGAAGATGCGGCTGACTGT
GADPH_exon	for	CTGGGCTACACTGAGCACC
	rev	AAGTGGTCGTTGAGGGCAATG
BCL2A1_promoter	for	CCCGAGTAGCTGGGATTACA
	rev	CCTAGCACTTTGGGAGGACA
BCL2A1_exon	for	AGTGCTACAAAATGTTGCGTTC
	rev	GGCAATTTGCTGTCGTAGAAGTT
HIST1H2AC_EXON1 (1)	for	CGTGAGCTTAGGCCGCTGGTTTT
	rev	CGCGAGCTTTGCCTCCTTGCTTA
HIST1H2AC_EXON1 (2)	for	GCTTAGGCCGCTGGTTTTGGTGA
	rev	AACTGGAGACCAGCGCGAGAAGA
HIST1H2AC_EXON2 (1)	for	GCTCTGCTATTCTGCTGGAATGGTG
	rev	ACGTTGAGTCTAGCCCAAGAGTGAC
HIST1H2AC_EXON2 (2)	for	CTCTGCTATTCTGCTGGAATGGTGG
	rev	CGTTGAGTCTAGCCCAAGAGTGAC
HIST1H2AD_EXON1 (1)	for	CCGAGCTAAGGCTAAGACCCGCT
	rev	GACTCGCTCGGAGTAGTTGCCCT
HIST1H2AD_EXON1 (2)	for	CGGAAAGGCCCGAGCTAAGGCTA
	rev	CTCGGCGGTCAGGTACTCCAACA
HIST1H2AD_EXON2 (1)	for	TCTGCGTTTTCAGAGCTCGGCG
	rev	TCACTCGCTTGGCGTGAATGGC
HIST1H2AD_EXON2 (2)	for	TCGCAAACTGCCATTCCAGCGT
	rev	TCACCGCCGAGCTCTGAAAACG
HIST1H2AE_EXON1 (1)	for	GGAAGCCACTATGTCTGGACGTGGA
	rev	GAACGCGTTTTAGCTTTTGCCCGAG
HIST1H2AE_EXON1 (2)	for	GCCACTATGTCTGGACGTGGAAAGC
	rev	AAACTGAAGACCGGCCCTGGAAGAA
HIST1H2AB_EXON1 (1)	for	GCAAACAAGGCGGTAAAGCTCGC
	rev	TGCAAACCTGCACGAGAAGACCG
HIST1H2AB_EXON1 (2)	for	TTTGCAGTTTCCTGTGGGCCGAG
	rev	TACTCAAGCACCGCCGCGAGATA
**Mouse Primers**		
H2AFJ_EXON1	for	GGCGAGGGACAACAAGAAGA
	rev	TCTGTCGGGTTCACTTGCTC
HIST3H2A_EXON1	for	GAAGATGTCTGGTCGTGGCA
	rev	TATTCTAGCACAGCCGCCAG
HIST1H2AB_EXON1	for	AACGCATACATGTCTGGACGC
	rev	CGAGTAGTTGCCCTTGCGG
GAPDH	for	TTCACCACCATGGAGAAGGC
	rev	GGCATGGACTGTGGTCATGA
PPIA	for	ATGGCAAATGCTGGACCAAA
	rev	GCCTTCTTTCACCTTCCCAAA
